# Desynchronization of Circadian Clocks in Cancer: A Metabolic and Epigenetic Connection

**DOI:** 10.3389/fendo.2017.00136

**Published:** 2017-06-19

**Authors:** Kiran Padmanabhan, Marc Billaud

**Affiliations:** ^1^“Molecular and Epigenetic Regulation of Biological Clocks”, Université de Lyon, Institut de Génomique Fonctionnelle de Lyon, CNRS UMR 5242, École Normale Supérieure de Lyon, Université Claude Bernard Lyon 1, Lyon, France; ^2^INSERM, Paris, France; ^3^“Clinical and Experimental Model of Lymphomagenesis”, Université de Lyon, Université Claude Bernard Lyon 1, INSERM 1052, CNRS 5286, Centre Léon Bérard, Centre de recherche en cancérologie de Lyon, Lyon, France

**Keywords:** circadian clocks, epigenetics, metabolomics, hematological malignancies, AMPK-MYC-SIRT axis

## Abstract

Circadian clocks are innate oscillators that drive daily rhythms in metabolism, physiology, and behavior. 24-h rhythms in gene expression, driven by core clock transcription factors, reflect the epigenetic state of the cell, which in turn is dictated by the metabolic environment. Cancer cells alter their metabolic state and gene expression and therefore are likely to tweak circadian clock function in their favor. Over the past decade, we have witnessed an extraordinary increase in systems-level studies that suggest intricate mechanistic links between the cellular metabolome and the circadian epigenome. In parallel, reprogramming of cellular clock function in cancers is increasingly evident and the role of clock genes in the development of hematological tumors, as well as their pathophysiological effects on tissues distal to the tumor, has been described. Furthermore, the interplay between components of the circadian clock, metabolic enzymes, and oncogenes is starting to be better understood, such as the close association between overexpression of the Myc oncogene and perturbation of circadian and metabolic rhythms, thus opening new avenues to treat cancers. This review article explores current knowledge on the circadian metabolome and the molecular pathways they control, with a focus on their involvement in the development of hematopoietic malignancies.

## Circadian Clocks

Circadian clocks are ubiquitous timers that synchronize physiology, metabolism, and behavior with the solar day. Cell-autonomous circadian oscillators drive daily rhythms in gene expression and protein function and help to build and maintain a homeostatic relationship with the external environment. Over two decades of genetic and proteomic screens have helped identify many molecular components of the circadian clock that seems to be conserved across species (1). In mammals, the master clock resides in the suprachiasmatic nucleus (SCN) within the hypothalamus. However, circadian clocks are not just restricted to the brain but instead are present virtually in all peripheral tissues and cells (2). Circadian phenomena emerge from self-sustained cellular clocks in these individual tissues and are globally sensitive to environmental rhythms in light and temperature and to social phenomena such as work, physical activity, and feeding behavior. SCN clocks keep track of external time mainly *via* photic cues from the retina and downstream neurohumoral pathways then synchronize oscillators in other brain nuclei as well as peripheral tissues (3). While the photoperiod serves as the dominant time-setting cue (*Zeitgeber, time giver*), time of food intake and daily rhythms in temperature are also important Zeitgebers. Food intake timing has physiological impact on sleep-activity rhythms, hormonal cycles, glucose homeostasis, and lipid metabolism and is deeply linked to organismal metabolic state (4). Consequences of modern lifestyle, such as excess light exposure at night, improper sleep schedules, high-fat diets, and intermittent feeding schedules, have a significant impact on normal circadian rhythms and its regulation of physiology and human health. Large sections of the human population that experience such desynchronous environments—night shift workers or regular long distance travelers, for example—display metabolic imbalances, with higher incidences of obesity and higher risks for cardiovascular disease and cancer (5, 6). In this review, we will highlight the cross talk between the cellular metabolic state and the circadian oscillator, in normal as well as in malignant conditions.

Within mammalian cells, a BMAL1:CLOCK transcription factor heterodimer drives the expression of the repressor complex component genes *PER* and *CRY*, which assemble into a ~1 MDa complex with other proteins to repress BMAL1:CLOCK function on chromatin (7–9). This negative feedback inhibits new *PER* and *CRY* synthesis. Eventually PER and CRY protein concentrations decline *via* turnover, resulting in the termination of negative feedback and reinitiation of a new molecular cycle. The negative feedback loop operates in almost all cells and tissues to generate stable, self-sustaining molecular rhythms with an intrinsic period close to 24 h. BMAL1:CLOCK *via* recruitment to promoter E-box elements (CACGTT) drives the rhythmic transcription of an additional 10–15% of expressed genes—the so-called clock-controlled genes (CCGs) (1, 10–13). A second regulatory loop comprising orphan nuclear receptors acts to stabilize the core oscillator. REV-ERBα, a nuclear hormone receptor under circadian transcriptional control, competes with retinoic acid-related orphan receptor alpha (RORα) to bind response elements (ROREs) in the *BMAL1* promoter. RORs activate transcription of *BMAL1*, whereas REVERBs represses it, effectively buffering BMAL1 levels and thereby stabilizing circadian transcription in individual cells (14). These pathways eventually impinge on regulation of rhythmic behavior and metabolism (15). These *cis*-acting elements, the E-boxes and ROREs, together help to promote precisely phased circadian transcription of clock output genes throughout the genome. The past few years have expanded the output pathways beyond the coding genome to a large fraction of the non-coding genome as well as the metabolome (10, 13, 16).

## Circadian Epigenetic Regulation and Metabolic Control

Studies over the past decade have clearly shown that epigenetic control plays a central role in circadian gene expression rhythms (17, 18). The mechanisms include methylation, acetylation, and other covalent modifications of histones, methylation of DNA itself, and posttranscriptional regulation of coding and non-coding RNA reviewed in great detail elsewhere (19, 20).

Genome-wide chromatin immunoprecipitation-deep sequencing analyses (ChIP-Seq) have demonstrated widespread epigenetic modifications that coincide with RNA PolII recruitment and rhythmic transcription in mammalian tissues (10, 13). A variety of enzymes that acetylate or methylate histones or conversely deacetylate or demethylate them have been found to interact with the core clock machinery (20). CLOCK itself was found to harbor histone H3 acetyltransferase activity targeting the H3K9 and H3K14 residues (21). Interestingly, CLOCK can trans-acetylate its binding partner BMAL1 (22).

The acetylation of cellular proteins (histones and non-histone proteins) depends on availability of acetyl-coA, the metabolite that provides the acetyl moiety. Nuclear and cytosolic acetyl-coA levels are controlled by a synthetase (acetyl-CoA synthetase, AceCS1) as well as ATP-citrate lyase (23). Cellular acetate levels also depend on the feeding state of the animal, the gut microbiome, as well as the activity of histone deacetylases (HDACs)—a class of proteins that play a critical role in transcriptional control. The class III HDAC SIRT1 drives circadian oscillations in acetyl-CoA levels by deacetylating and activating AceCS1, and impacts several metabolic pathways including autophagy (24).

Unlike class I and II HDACs, the activity of the class III HDACs (e.g., SIRT1, SIRT6) depends on yet another metabolite—NAD. A series of reports initially identified a crucial role for nicotinamide adenine dinucleotide (NAD^+^) in clock control and later went on to describe the role of NAD-dependent deacetylases, SIRT1 and SIRT6 circadian physiology (25–32). The levels of nicotinamide adenine nucleotide (NAD^+^), an essential co-factor for these Sirtuins, shows robust 24-h rhythms (31). SIRTs consume NAD for their activity and generate nicotinamide adenine mononucleotide (NAM), which acts as a product inhibitor for this enzymatic reaction (33). In the salvage pathway, NAM is converted *via* a mononucleotide intermediate (NMN) back to NAD by mononucleotide adenylyltransferases. NMN formation is regulated by NAMPT whose expression is under circadian control, thus ultimately resulting in circadian oscillations in NAD synthesis (34). Direct regulation of the NAMPT pathway also occurs *via* the AMP-activated kinase (AMPK) pathway, which senses AMP/ATP ratio and is key in adapting energetic supply to the nutrient demands of cells facing situations of metabolic stress. AMPK regulates the levels of NAD^+^ by increasing NAMPT expression and thus activates SIRT1 pathway (35, 36).

SIRT1 eventually drives rhythmic deacetylation of histone H3, BMAL1, and PER2 (26). Specific loss of SIRT1 in murine livers indicates that along with SIRT6, it contributes to genomic partitioning of circadian transcription (28). Meanwhile, AMPK can integrate metabolic cues to modulate the clock circuitry and the circadian remodeling of chromatin. AMPK promotes the degradation of the circadian repressors CRY1 and PER2 (37, 38) while it activates SIRT1 (35, 36).

Thus, not only do epigenetic pathways depend on the oscillating metabolome but synthesis and salvage pathways that generate these metabolites are also regulated by cell-autonomous clocks. In this regard, the Sirtuins (along with its regulators such as the AMPK pathway) seem to be central to the modulation of the circadian epigenome and likely culprits in metabolic disorders stemming from clock dysfunction.

## Circadian Regulation of the Metabolome

Cellular metabolites not only influence cellular state but also are a direct readout of the biochemical activity of a cell. Unlike transcriptomic or epigenomic methods, metabolomic approaches allow for a global quantitative assessment of endogenous metabolites within a biologic system, thus reflecting changes in cellular phenotype. Clocks regulate multiple aspects of animal metabolism and “systems” approaches have revealed widespread cross talk between 24hr rhythms and metabolic pathways. Accordingly, a recent study revealed that 50% of mouse liver metabolites are circadian (39). Sleep deprivation in animal models strongly impact glucose and energy metabolism (40–43). By comparing wild-type and *Clock*^−/−^ mice, Eckel-Mahan and coworkers demonstrated that the diurnal metabolome and rhythms in enzymes controlling pyrimidine salvage pathway, Kreb’s cycle and lipid metabolism are controlled by environmental signals. *Clock* mutant mice, in contrast, show very few rhythmic metabolites (44). Ultimately, the timing of food intake and nutritional richness seem to underlie many physiological consequences of the circadian metabolome. High-fat diets lead to widespread reorganization of metabolic pathways, reduction in BMAL1:CLOCK recruitment *via* misregulation of the PPARγ pathway (18). Time-restricted feeding was shown to promote healthy physiology, robust circadian rhythms, and protects against obesity and metabolic consequences of a high-fat diet (45, 46). Of late, the role of the gut microbiota as a mediator of nutritional state to set tissue clocks has been extensively reported (47–50).

## Cancer Metabolism: The Case of Blood Tumors

When malignancy arises, these homeostatic mechanisms are clearly lost or altered, as the energy needs of a cancer cell are vastly different. Tumor development is coincident with massive genomic and metabolic reprogramming with fundamental changes to epigenetic state. One of the earliest described metabolic hallmarks of tumors was an elevated glycolytic rate even in the presence of sufficient oxygen. This so-called Warburg effect or aerobic glycolysis is now known to constitute an adaptation of malignant cells to their nutrient-poor microenvironment in order to optimize the uptake of these nutrients to synthesize macromolecules needed for their survival and proliferation (51, 52). Tumors reprogram their metabolism to ensure a steady supply of metabolites to generate new biomass. Thus, malignancy is coincident with elevated aerobic glycolysis, increased lipogenesis needed for membrane production, fatty acid oxidation, and a dependency on methionine synthesis—pathways that clearly display diurnal or circadian rhythms. Hematological malignancies account for nearly 10% of clinical diagnoses of all cancers and their metabolic signature has been reviewed recently in depth (53). Nearly 30 metabolites were found to be differentially expressed in acute lymphoid leukemia (ALL) patients, glycerophospholipid metabolism in particular seemed to be linked to the development and disease progression. In acute myeloid leukemia (AML), UDP-d-glucose, a glycogenic precursor, was found to be persistently upregulated (54). Chronic lymphocytic leukemia (CLL) patients on the other hand showed high levels of pyruvate and glutamate in their blood. Myelodysplastic syndromes following therapy were correlated with alterations in the metabolism of aspartate, alanine, dicarboxylate, glyoxylate, and phenylalanine (55). Given the widespread circadian influence on the metabolite levels, care needs to be taken in interpretation of tumor metabolome data and circadian biomarkers could potentially be used to normalize noise in these studies (56, 57).

## Clock Genes and Circadian Misregulation in Hematopoietic Malignancies

Not surprisingly, core clock gene dysfunction has been implicated in many hematological malignancies. Diffuse large B-cell lymphoma (DLBCL), ALL, and AML display promoter hypermethylation at the *BMAL1* gene. The CCAAT/enhancer binding protein alpha (C/EBPalpha) regulates the expression of *PER2* and depletion of BMAL1 in unmethylated cells promotes tumor growth while its reintroduction in tumor cells slowed down growth in colony assays and nude mice (58). The mixed-lineage leukemia genes MLL1 and MLL3 are CCGs that regulate recruitment of BMAL1:CLOCK to chromatin by controlling promoter histone methylations and furthermore epigenetically regulate the function of many downstream target genes rhythmically over 24 h (59–61). In CLL patients, CRY1 expression or a *CRY1*:*PER*2 expression ratio could isolate subgroups of patients and help prognosis (62). PER2 was found to be reduced in lymphoma and AML patient samples while overexpression of PER2 led to cell cycle arrest and loss of clonogenicity (63). In a small study, newly diagnosed chronic myeloid leukemia (CML) patients showed disruption in daily core clock gene expression patterns, which were then partially reversed in those that displayed cytogenetic and molecular response to imatinib treatment (64). The tumor suppressor PML has been shown to directly interact with PER2 and regulate its nuclear localization (65). Double *PML/PER* knockout mice showed changes in circadian behavior implying a reciprocal role for PML in clock function (66). While most studies point to an oncogenic role for clock mutations, there are exceptions. Recently, Puram et al. described how BMAL1:CLOCK are essential transcription factors in a murine model of AML with a well-defined leukemia stem cell population (67). Disruption of the canonical clock pathway in this study surprisingly produced antileukemic effects and impaired proliferation, increased differentiation, and a depletion of LSCs. Thus, while it is evident that clock genes play a role in hematological malignancies, whether they promote or hamper tumor growth is not clear. Indeed, the effectors of the core clock machinery might also display non-circadian functions, which would be involved in the cancer development, as exemplified by BMAL1 function during early development (68). A potential link and a resolution to this apparent dilemma could lie in a series of recent studies that have explored the role of the oncogene MYC in circadian clocks.

## MYC—A Potential Link Between Hematological Malignancies and Clocks

MYC, a helix-loop-helix leucine zipper transcription factor, was originally identified as the genetic target of the t(8;14)(q24;q32) chromosome translocation in Burkitt lymphoma (69). *MYC* expression is deregulated in a wide array of cancers and is generally associated with poor prognosis. Decades of work in cancer biology has linked Myc activation to the loss of cell cycle regulation, in the activation of the p53 pathway *via* inhibition of MDM2-mediated degradation and in promoting pro-apoptotic pathways *via* the induction of BIM-BAX-BAK cascade in mitochondria (69). In lymphomas, MYC affects the metabolome, by stimulating glycolysis and glutaminolysis (70). Increased *MYC* expression in normal cells can drive both enhanced cellular proliferation and also conversely lead to upregulation of pro-apoptotic pathways, reflecting a need for tight regulation of its expression in normal cells.

MYC and BMAL1:CLOCK bind to identical DNA cis-elements and much like the latter, MYC is thought to regulate the transcription of 10–15% of genes in the human genome. Its misexpression and activation in cancer cells is thought to lead to large-scale transcriptomic effects. At high oncogenic levels, MYC potentially could bind and compete for BMAL1:CLOCK E-boxes and disrupt circadian transcription. Instead, Shostak et al. found that overexpression of *MYC* has little effect on transcription of *Per* and *Cry* genes. High levels of MYC *via* the assembly of a repressive MYC–MIZ1 complex led to the downregulation of BMAL1 and CLOCK expression and lowered protein levels (71). Another study linked the loss of BMAL1 expression to the MYC-driven upregulation of REV-ERBα and REV-ERBβ, secondary oscillator loop components that dampen BMAL1 expression (72). BMAL1 levels correlate inversely with that of MYC protein in nearly 100 human lymphomas (71). Together these studies point to two different means by which Myc impacts circadian clock dynamics. A study from Tyler Jacks’ group offered further support for a circadian link to Myc function—wherein either physiologic disruption of clocks (using a jetlag protocol) or genetic loss of central clock components led to increased *c-MYC* expression, loss of metabolic homeostasis, and increased proliferation in a lung adenocarcinoma model (73). c-MYC expression can be regulated by E-boxes in its own promoter and by core clock proteins (74, 75). Additionally, it can also be regulated at the posttranslational level (76), with a very recent study indicating that the circadian repressor protein CRY2 targets MYC for degradation *via* the F-box protein FBXW7 (77). Thus, a negative cross talk between the MYC pathway and circadian oscillator is likely to exist in cancer cells.

*Could SIRT1 then be the link between circadian clock dysfunction and Myc-driven cancers?* Intriguingly, several studies have explored the cross talk between the SIRT1 and MYC pathways with some reports indicating a positive feedback between the two pathways (78). A c-MYC-related network that enhances SIRT1 protein expression in human AML LSCs was found to contribute to leukemic stem cell maintenance (79). In some instances, increased expression of c-MYC leads to increase in SIRT1 protein, NAD, and NAMPT mRNA and additionally leads to SIRT1 activation by sequestering the inhibitory protein deleted in breast cancer 1 (80). SIRT1 in turn stabilizes MYC levels and promotes its transcriptional activity. NAMPT thus is a target of both BMAL1:CLOCK and oncogenic MYC in Myc-driven cancers. While a series of studies have shown a positive feedback loop existing between MYC and SIRT1 function in cells, one report indicated negative feedback between these two pathways (81). The contrasting data might result from variable SIRT1 protein levels in cells. SIRT1 dosage was shown to be critical in the regulation of Myc, with non-overlapping results obtained upon either heterozygous or homozygous deletion of the SIRT1 gene (82). In the study by Ren et al., heterozygous deletion of *SIRT1* induced c-MYC expression, enhancing glutamine metabolism and subsequent proliferation and malignancy, while SIRT1 homozygous deletion triggered apoptosis and reduced cancer formation. The impact on cellular clocks under these distinct and opposing states of SIRT1 expression and MYC function however remains to be explored.

Finally, much like MYC, AMPK functions as a conditional tumor suppressor and as a contextual oncogene—thus, a dysfunction of the LKB1/AMPK signaling pathway could rewire the circadian epigenome and rhythmic gene expression to contribute eventually to cancer development. AMPK functions in parallel with MYC (83) and it is a therapeutic target in several hematopoietic malignancies including AML (84), ALL (85), CLL (86), lymphomas (87, 88), and MM (89). Moreover, Metformin, a known activator of AMPK, modulates the metabolic rhythms generated by the circadian clock (90). It will thus be relevant to explore whether the antitumor effects of pharmacological agents targeting AMPK depend on their ability to normalize daily rhythms through the synchronization of the clock involving the reshaping of the circadian chromatin landscape and the regulation of the MYC-SIRT1 axis.

## Summary

**Figure 1:**
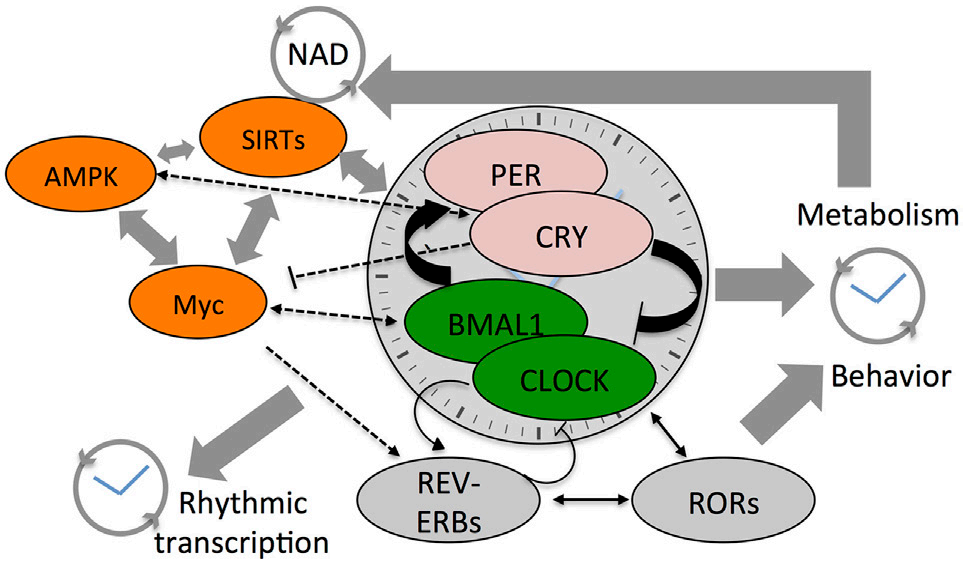
Cartoon depicts the cross talk between various components of the core clock mechanism and the stabilizing loops (Rev-erbs and RORs) that drive daily system-wide rhythms in gene expression and metabolites. Also shown are the three core-metabolic sensing pathways—AMP-activated kinase (AMPK), Myc, and SIRTs. NAD^+^ feedsback to control the clock by regulating the function of class III histone deacetylases—the Sirtuins. The SIRTs and core clock components regulate Myc expression that competes with BMAL1:CLOCK on transcription targets sites and also drives Rev-erb expression. Colored components within the cartoon highlight the factors linked to hematological malignancies such as acute myeloid leukemia, chronic myeloid leukemia, and diffuse large B-cell lymphoma.

Circadian architecture regulates cellular metabolic state (see Figure 1). Far from being an unidirectional regulatory event, circadian transcription that drives oscillations in small metabolites is in turn affected by the abundance of these small molecules. While homeostasis with the environment ensures a regular rhythm in metabolite levels, perturbations that arise from modern lifestyles have now all been shown to alter rhythmic pathways. Many malignancies of hematological origin, which account for nearly 10% of clinical diagnoses of all cancers, display altered clock function and parallel widespread metabolic arrhythmia. MYC is either amplified or shows misexpression in at least 50% of all cancers and a majority of human leukemia and lymphoma cases (91) and SIRT1 is implicated in similar hematological malignancies (92, 93). These results suggest that disruption of circadian clocks in blood cancers might be more widespread than currently appreciated. Circadian metabolomic screens, when integrated with transcriptomics and epigenomics may thus provide a more comprehensive understanding of the complex biology associated with tumorigenesis and malignant transformation.

## Author Contributions

KP conceived, drafted, and prepared the manuscript along with MB.

## Conflict of Interest Statement

The authors declare that the research was conducted in the absence of any commercial or financial relationships that could be construed as a potential conflict of interest.
